# (2,4-Di-*tert*-butyl-6-{(*E*)-[(*E*)-2-(2-methoxy­benzyl­ideneamino)cyclo­hexyl]imino­meth­yl}phenolato)dimethyl­aluminum(III)

**DOI:** 10.1107/S1600536808041858

**Published:** 2008-12-17

**Authors:** Jin-Cai Wu

**Affiliations:** a College of Chemistry and Chemical Engineering, State Key Laboratory of Applied Organic Chemistry, Lanzhou University, Lanzhou 730000, People’s Republic of China

## Abstract

The title compound, [Al(CH_3_)_2_(C_29_H_39_N_2_O_2_)], exhibits disorder of one of the *tert*-butyl groups on the Schiff base ligand, where each methyl group is located over two sites, with occupancy factors of 0.57 (1) and 0.43 (1). The geometry around the Al^III^ atom is distorted tetra­hedral, defined by two methyl groups, one N and one O atom from the Schiff base ligand.

## Related literature

For general background, see: Endo *et al.* (1987 ▶); Wu *et al.* (2006 ▶).
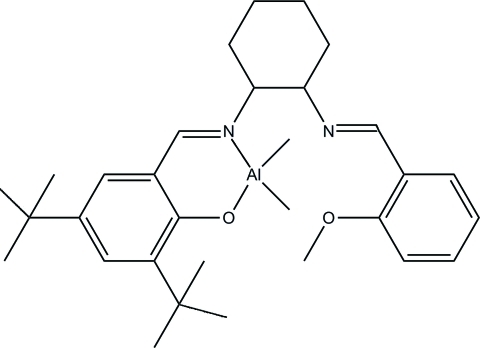


## Experimental

### 

#### Crystal data

[Al(CH_3_)_2_(C_29_H_39_N_2_O_2_)]
*M* *_r_* = 504.67
Monoclinic, 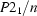

*a* = 15.5841 (9) Å
*b* = 10.4430 (6) Å
*c* = 20.4534 (12) Å
β = 111.810 (1)°
*V* = 3090.4 (3) Å^3^
*Z* = 4
Mo *K*α radiation
μ = 0.09 mm^−1^
*T* = 303 (2) K
0.32 × 0.30 × 0.25 mm

#### Data collection

Bruker SMART 1K CCD area-detector diffractometer
Absorption correction: multi-scan (*SADABS*; Sheldrick, 1996 ▶) *T*_min_ = 0.968, *T*_max_ = 0.979
16414 measured reflections
6069 independent reflections
4158 reflections with *I* > 2σ(*I*)
*R*_int_ = 0.024

#### Refinement

*R*[*F* ^2^ > 2σ(*F* ^2^)] = 0.045
*wR*(*F* ^2^) = 0.140
*S* = 1.07
6069 reflections
353 parameters
H-atom parameters constrained
Δρ_max_ = 0.21 e Å^−3^
Δρ_min_ = −0.23 e Å^−3^

### 

Data collection: *SMART* (Bruker, 2007 ▶); cell refinement: *SAINT* (Bruker, 2007 ▶); data reduction: *SAINT*; program(s) used to solve structure: *SHELXS97* (Sheldrick, 2008 ▶); program(s) used to refine structure: *SHELXL97* (Sheldrick, 2008 ▶); molecular graphics: *SHELXTL* (Sheldrick, 2008 ▶); software used to prepare material for publication: *SHELXTL*.

## Supplementary Material

Crystal structure: contains datablocks I, global. DOI: 10.1107/S1600536808041858/hy2172sup1.cif

Structure factors: contains datablocks I. DOI: 10.1107/S1600536808041858/hy2172Isup2.hkl

Additional supplementary materials:  crystallographic information; 3D view; checkCIF report

## Figures and Tables

**Table 1:** Selected bond lengths (Å)

Al1—O1	1.7497 (13)
Al1—N1	1.9705 (14)
Al1—C31	1.936 (3)
Al1—C30	1.950 (3)

**Fig. 1.**
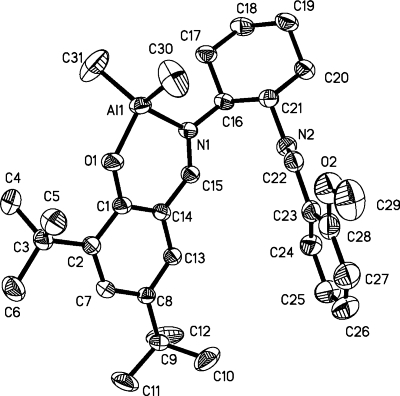
Molecular structure of the title compound. Displacement ellipsoids are drawn at the 30% probability level. H atoms have been omitted for clarity. The minor positions of the disordered methyl groups are not shown.

## supplementary crystallographic information

### Comment

In past decades, significant advance has been made in polymerization of cyclic esters, such as poly-ε-caprolactone (Endo *et al.*, 1987) and polylactide (Wu *et al.*, 2006). A particularly convenient method for the synthesis of polylactides is the ring-opening polymerization (ROP) of lactides. Due to the advantages of well controlled molecular weight and low polydispersity, many metal complexes have been used to polymerize lactides through ROP process. Here, we report a new aluminium complex, which can be used for the investigation of ROP of lactide.

In the title compound, the Al^III^ atom is coordinated in a tetrahedral coordination geometry by two methyl groups, one O atom and one N atom from the Schiff base ligand (Fig. 1). The Al1—O1 distance of 1.7497 (13) Å is shorter than that of Al1—N1 [1.9705 (14) Å], while two Al—C distances are almost identical (Table 1).

### Experimental

The title aluminium complex was prepared as following. The Schiff base ligand (1 mmol) was dissolved in toluene (20 ml), and then trimethylaluminum (1.2 mmol) in hexane solution (0.6 ml, 2*M*) was added slowly. The mixture was stirred and refluxed for 5 h. Then volatile materials were removed under vacuum. The residue was recrystallized in toluene to give yellow crystalline solid (yield: 92%, 0.46 g).

### Refinement

H atoms were positioned geometrically and refined using a riding model, with C—H = 0.93 (aromatic), 0.98 (CH), 0.97 (CH_2_) and 0.96 (CH_3_) Å and with *U*_iso_(H) = 1.2(1.5 for methyl group)*U*_eq_(C). One of the *tert*-butyl groups shows a positional disorder over two sites, with occupancy factors of 0.57 (1) and 0.43 (1), respectively.

### Crystal data

**Table:**

[Al(CH_3_)_2_(C_29_H_39_N_2_O_2_)]	*F*(000) = 1096
*M**_r_* = 504.67	*D*_x_ = 1.085 Mg m^−^^3^
Monoclinic, *P*2_1_/*n*	Mo *K*α radiation, λ = 0.71073 Å
Hall symbol: -P 2yn	Cell parameters from 16999 reflections
*a* = 15.5841 (9) Å	θ = 2.4–25.8°
*b* = 10.4430 (6) Å	µ = 0.09 mm^−^^1^
*c* = 20.4534 (12) Å	*T* = 303 K
β = 111.810 (1)°	Block, yellow
*V* = 3090.4 (3) Å^3^	0.32 × 0.30 × 0.25 mm
*Z* = 4	

### Data collection

**Table:**

Bruker SMART 1K CCD area-detector diffractometer	6069 independent reflections
Radiation source: fine-focus sealed tube	4158 reflections with *I* > 2σ(*I*)
graphite	*R*_int_ = 0.024
φ and ω scans	θ_max_ = 26.0°, θ_min_ = 2.1°
Absorption correction: multi-scan (*SADABS*; Sheldrick, 1996)	*h* = −18→19
*T*_min_ = 0.968, *T*_max_ = 0.979	*k* = −12→12
16414 measured reflections	*l* = −25→19

### Refinement

**Table:**

Refinement on *F*^2^	Primary atom site location: structure-invariant direct methods
Least-squares matrix: full	Secondary atom site location: difference Fourier map
*R*[*F*^2^ > 2σ(*F*^2^)] = 0.045	Hydrogen site location: inferred from neighbouring sites
*wR*(*F*^2^) = 0.140	H-atom parameters constrained
*S* = 1.07	*w* = 1/[σ^2^(*F*_o_^2^) + (0.0758*P*)^2^ + 0.13*P*] where *P* = (*F*_o_^2^ + 2*F*_c_^2^)/3
6069 reflections	(Δ/σ)_max_ = 0.001
353 parameters	Δρ_max_ = 0.21 e Å^−^^3^
0 restraints	Δρ_min_ = −0.23 e Å^−^^3^

### Fractional atomic coordinates and isotropic or equivalent isotropic displacement parameters (Å^2^)

**Table:**

	*x*	*y*	*z*	*U*_iso_*/*U*_eq_	Occ. (<1)
Al1	0.15093 (4)	−0.23257 (5)	1.01539 (3)	0.05346 (17)	
N1	0.16403 (9)	−0.06832 (12)	1.06560 (7)	0.0453 (3)	
N2	0.33233 (10)	0.04471 (14)	1.15816 (8)	0.0548 (4)	
O1	0.13324 (10)	−0.16488 (11)	0.93314 (6)	0.0679 (4)	
O2	0.54897 (11)	−0.04008 (19)	1.10861 (9)	0.0930 (5)	
C1	0.13070 (12)	−0.04527 (15)	0.91126 (9)	0.0484 (4)	
C2	0.10964 (11)	−0.01667 (16)	0.83927 (8)	0.0467 (4)	
C3	0.08400 (13)	−0.12465 (17)	0.78423 (9)	0.0578 (5)	
C4	−0.00146 (15)	−0.1966 (2)	0.78482 (12)	0.0797 (6)	
H4A	−0.0522	−0.1379	0.7747	0.120*	
H4B	0.0118	−0.2341	0.8304	0.120*	
H4C	−0.0175	−0.2628	0.7498	0.120*	
C5	0.16522 (16)	−0.2184 (2)	0.80022 (12)	0.0764 (6)	
H5A	0.2185	−0.1733	0.7995	0.115*	
H5B	0.1490	−0.2848	0.7653	0.115*	
H5C	0.1789	−0.2558	0.8459	0.115*	
C6	0.06099 (19)	−0.0726 (2)	0.70960 (11)	0.0845 (7)	
H6A	0.0100	−0.0141	0.6982	0.127*	
H6B	0.0447	−0.1423	0.6767	0.127*	
H6C	0.1139	−0.0288	0.7072	0.127*	
C7	0.11354 (11)	0.11049 (16)	0.82141 (8)	0.0473 (4)	
H7A	0.1002	0.1293	0.7742	0.057*	
C8	0.13610 (11)	0.21307 (15)	0.86906 (8)	0.0443 (4)	
C9	0.14072 (12)	0.35157 (16)	0.84619 (9)	0.0514 (4)	
C10	0.2401 (4)	0.4026 (6)	0.8825 (5)	0.103 (3)	0.567 (11)
H10A	0.2818	0.3511	0.8690	0.155*	0.567 (11)
H10B	0.2573	0.3987	0.9327	0.155*	0.567 (11)
H10C	0.2429	0.4897	0.8685	0.155*	0.567 (11)
C11	0.1165 (8)	0.3676 (7)	0.7679 (3)	0.126 (4)	0.567 (11)
H11A	0.1560	0.3140	0.7531	0.189*	0.567 (11)
H11B	0.1250	0.4554	0.7578	0.189*	0.567 (11)
H11C	0.0532	0.3435	0.7431	0.189*	0.567 (11)
C12	0.0790 (6)	0.4378 (5)	0.8707 (6)	0.115 (4)	0.567 (11)
H12A	0.0933	0.4249	0.9201	0.173*	0.567 (11)
H12B	0.0154	0.4168	0.8449	0.173*	0.567 (11)
H12C	0.0897	0.5258	0.8623	0.173*	0.567 (11)
C10'	0.0423 (4)	0.3982 (7)	0.8113 (7)	0.091 (3)	0.433 (11)
H10D	0.0158	0.3635	0.7646	0.137*	0.433 (11)
H10E	0.0417	0.4900	0.8089	0.137*	0.433 (11)
H10F	0.0067	0.3706	0.8383	0.137*	0.433 (11)
C11'	0.1909 (10)	0.4354 (7)	0.9073 (4)	0.128 (6)	0.433 (11)
H11D	0.2562	0.4205	0.9220	0.191*	0.433 (11)
H11E	0.1712	0.4162	0.9455	0.191*	0.433 (11)
H11F	0.1778	0.5235	0.8939	0.191*	0.433 (11)
C12'	0.1836 (8)	0.3528 (8)	0.7914 (7)	0.105 (4)	0.433 (11)
H12D	0.2182	0.2754	0.7947	0.157*	0.433 (11)
H12E	0.2241	0.4252	0.7990	0.157*	0.433 (11)
H12F	0.1358	0.3585	0.7454	0.157*	0.433 (11)
C13	0.15151 (11)	0.18289 (16)	0.93786 (8)	0.0472 (4)	
H13A	0.1647	0.2484	0.9709	0.057*	
C14	0.14805 (11)	0.05669 (15)	0.95983 (8)	0.0458 (4)	
C15	0.16173 (12)	0.03794 (16)	1.03283 (9)	0.0484 (4)	
H15A	0.1700	0.1119	1.0598	0.058*	
C16	0.18522 (11)	−0.05630 (16)	1.14241 (8)	0.0476 (4)	
H16A	0.1626	0.0273	1.1508	0.057*	
C17	0.13655 (13)	−0.15877 (18)	1.16847 (9)	0.0590 (5)	
H17A	0.1552	−0.2426	1.1582	0.071*	
H17B	0.0703	−0.1510	1.1437	0.071*	
C18	0.15928 (14)	−0.1471 (2)	1.24726 (10)	0.0697 (6)	
H18A	0.1333	−0.0681	1.2568	0.084*	
H18B	0.1313	−0.2178	1.2628	0.084*	
C19	0.26296 (15)	−0.1478 (2)	1.28832 (10)	0.0718 (6)	
H19A	0.2752	−0.1326	1.3378	0.086*	
H19B	0.2877	−0.2313	1.2841	0.086*	
C20	0.31094 (14)	−0.04610 (19)	1.26148 (10)	0.0642 (5)	
H20A	0.3772	−0.0523	1.2869	0.077*	
H20B	0.2912	0.0378	1.2706	0.077*	
C21	0.28978 (12)	−0.05982 (16)	1.18267 (9)	0.0500 (4)	
H21A	0.3142	−0.1417	1.1738	0.060*	
C22	0.39372 (12)	0.01774 (18)	1.13415 (9)	0.0546 (4)	
H22A	0.4110	−0.0675	1.1340	0.065*	
C23	0.43922 (12)	0.1148 (2)	1.10646 (9)	0.0595 (5)	
C24	0.40447 (16)	0.2387 (2)	1.09247 (11)	0.0711 (6)	
H24A	0.3513	0.2601	1.1005	0.085*	
C25	0.4475 (2)	0.3302 (3)	1.06683 (13)	0.0946 (8)	
H25A	0.4241	0.4130	1.0580	0.113*	
C26	0.5259 (2)	0.2971 (4)	1.05458 (15)	0.1100 (10)	
H26A	0.5550	0.3583	1.0371	0.132*	
C27	0.56172 (18)	0.1753 (3)	1.06771 (13)	0.0975 (9)	
H27A	0.6148	0.1548	1.0593	0.117*	
C28	0.51884 (14)	0.0838 (3)	1.09337 (11)	0.0739 (6)	
C29	0.63063 (16)	−0.0780 (3)	1.09781 (16)	0.1179 (11)	
H29A	0.6439	−0.1661	1.1110	0.177*	
H29B	0.6815	−0.0256	1.1261	0.177*	
H29C	0.6215	−0.0677	1.0490	0.177*	
C30	0.2676 (2)	−0.3257 (3)	1.05250 (15)	0.1228 (11)	
H30A	0.2614	−0.4058	1.0281	0.184*	
H30B	0.2834	−0.3416	1.1018	0.184*	
H30C	0.3154	−0.2757	1.0458	0.184*	
C31	0.0389 (2)	−0.3223 (3)	1.00777 (18)	0.1297 (13)	
H31A	0.0351	−0.4013	0.9829	0.194*	
H31B	−0.0138	−0.2700	0.9826	0.194*	
H31C	0.0396	−0.3400	1.0540	0.194*	

### Atomic displacement parameters (Å^2^)

**Table:**

	*U*^11^	*U*^22^	*U*^33^	*U*^12^	*U*^13^	*U*^23^
Al1	0.0741 (4)	0.0371 (3)	0.0544 (3)	−0.0002 (2)	0.0299 (3)	0.0044 (2)
N1	0.0535 (8)	0.0414 (7)	0.0434 (7)	0.0013 (6)	0.0210 (6)	0.0050 (6)
N2	0.0618 (9)	0.0534 (9)	0.0524 (8)	−0.0063 (7)	0.0247 (7)	−0.0034 (7)
O1	0.1125 (11)	0.0373 (7)	0.0512 (7)	−0.0068 (7)	0.0272 (7)	0.0008 (5)
O2	0.0696 (9)	0.1153 (14)	0.1041 (13)	0.0090 (9)	0.0437 (9)	−0.0042 (11)
C1	0.0595 (10)	0.0381 (9)	0.0472 (9)	−0.0011 (7)	0.0191 (8)	0.0038 (7)
C2	0.0512 (9)	0.0450 (9)	0.0422 (9)	0.0001 (7)	0.0154 (7)	0.0005 (7)
C3	0.0753 (12)	0.0492 (10)	0.0454 (10)	−0.0019 (9)	0.0181 (9)	−0.0044 (8)
C4	0.0861 (15)	0.0682 (14)	0.0754 (14)	−0.0189 (12)	0.0191 (12)	−0.0149 (11)
C5	0.0987 (16)	0.0602 (13)	0.0728 (14)	0.0131 (11)	0.0348 (12)	−0.0082 (10)
C6	0.1270 (19)	0.0718 (14)	0.0487 (11)	−0.0002 (13)	0.0255 (12)	−0.0085 (10)
C7	0.0511 (9)	0.0506 (10)	0.0389 (8)	0.0029 (7)	0.0153 (7)	0.0058 (7)
C8	0.0454 (8)	0.0409 (9)	0.0453 (9)	0.0003 (7)	0.0152 (7)	0.0061 (7)
C9	0.0591 (10)	0.0440 (9)	0.0494 (10)	0.0007 (8)	0.0183 (8)	0.0107 (8)
C10	0.080 (3)	0.059 (3)	0.145 (7)	−0.019 (2)	0.012 (3)	0.033 (4)
C11	0.232 (11)	0.071 (4)	0.055 (3)	−0.032 (6)	0.030 (5)	0.023 (2)
C12	0.155 (8)	0.055 (3)	0.184 (10)	0.040 (4)	0.119 (8)	0.047 (5)
C10'	0.086 (4)	0.057 (4)	0.137 (8)	0.022 (3)	0.049 (4)	0.049 (4)
C11'	0.189 (14)	0.053 (4)	0.080 (5)	−0.046 (6)	−0.020 (6)	0.021 (3)
C12'	0.149 (8)	0.064 (4)	0.143 (10)	0.006 (5)	0.102 (8)	0.039 (5)
C13	0.0559 (9)	0.0386 (9)	0.0451 (9)	−0.0005 (7)	0.0164 (8)	0.0005 (7)
C14	0.0551 (9)	0.0413 (9)	0.0396 (8)	−0.0004 (7)	0.0159 (7)	0.0029 (7)
C15	0.0601 (10)	0.0390 (9)	0.0454 (9)	0.0005 (7)	0.0188 (8)	−0.0001 (7)
C16	0.0590 (10)	0.0449 (9)	0.0426 (9)	0.0055 (8)	0.0234 (8)	0.0048 (7)
C17	0.0607 (11)	0.0639 (12)	0.0574 (11)	−0.0026 (9)	0.0279 (9)	0.0117 (9)
C18	0.0859 (14)	0.0747 (14)	0.0610 (12)	−0.0008 (11)	0.0415 (11)	0.0137 (10)
C19	0.0965 (15)	0.0705 (13)	0.0490 (11)	−0.0007 (11)	0.0278 (11)	0.0125 (10)
C20	0.0771 (13)	0.0640 (12)	0.0478 (10)	−0.0043 (10)	0.0188 (9)	0.0028 (9)
C21	0.0589 (10)	0.0446 (9)	0.0490 (10)	0.0004 (8)	0.0230 (8)	0.0020 (7)
C22	0.0536 (10)	0.0596 (11)	0.0482 (10)	−0.0019 (8)	0.0162 (8)	−0.0023 (8)
C23	0.0560 (10)	0.0767 (14)	0.0454 (10)	−0.0146 (9)	0.0183 (8)	−0.0072 (9)
C24	0.0807 (14)	0.0736 (14)	0.0599 (12)	−0.0143 (11)	0.0273 (10)	−0.0010 (10)
C25	0.113 (2)	0.0905 (18)	0.0806 (16)	−0.0268 (15)	0.0364 (15)	0.0052 (14)
C26	0.108 (2)	0.135 (3)	0.093 (2)	−0.052 (2)	0.0451 (17)	0.0098 (19)
C27	0.0746 (15)	0.144 (3)	0.0832 (17)	−0.0269 (17)	0.0405 (13)	0.0015 (17)
C28	0.0574 (12)	0.1061 (19)	0.0569 (12)	−0.0145 (12)	0.0197 (10)	−0.0073 (12)
C29	0.0671 (15)	0.173 (3)	0.119 (2)	0.0158 (17)	0.0409 (15)	−0.025 (2)
C30	0.141 (2)	0.109 (2)	0.0927 (19)	0.0628 (19)	0.0137 (17)	−0.0238 (16)
C31	0.165 (3)	0.112 (2)	0.156 (3)	−0.080 (2)	0.112 (2)	−0.063 (2)

### Geometric parameters (Å, °)

**Table:**

Al1—O1	1.7497 (13)	C10'—H10F	0.9600
Al1—N1	1.9705 (14)	C11'—H11D	0.9600
Al1—C31	1.936 (3)	C11'—H11E	0.9600
Al1—C30	1.950 (3)	C11'—H11F	0.9600
N1—C15	1.290 (2)	C12'—H12D	0.9600
N1—C16	1.486 (2)	C12'—H12E	0.9600
N2—C22	1.259 (2)	C12'—H12F	0.9600
N2—C21	1.459 (2)	C13—C14	1.400 (2)
O1—C1	1.3225 (19)	C13—H13A	0.9300
O2—C28	1.372 (3)	C14—C15	1.441 (2)
O2—C29	1.426 (3)	C15—H15A	0.9300
C1—C14	1.412 (2)	C16—C17	1.518 (2)
C1—C2	1.416 (2)	C16—C21	1.529 (2)
C2—C7	1.385 (2)	C16—H16A	0.9800
C2—C3	1.538 (2)	C17—C18	1.521 (3)
C3—C6	1.532 (3)	C17—H17A	0.9700
C3—C4	1.533 (3)	C17—H17B	0.9700
C3—C5	1.537 (3)	C18—C19	1.519 (3)
C4—H4A	0.9600	C18—H18A	0.9700
C4—H4B	0.9600	C18—H18B	0.9700
C4—H4C	0.9600	C19—C20	1.514 (3)
C5—H5A	0.9600	C19—H19A	0.9700
C5—H5B	0.9600	C19—H19B	0.9700
C5—H5C	0.9600	C20—C21	1.527 (2)
C6—H6A	0.9600	C20—H20A	0.9700
C6—H6B	0.9600	C20—H20B	0.9700
C6—H6C	0.9600	C21—H21A	0.9800
C7—C8	1.402 (2)	C22—C23	1.466 (3)
C7—H7A	0.9300	C22—H22A	0.9300
C8—C13	1.373 (2)	C23—C24	1.391 (3)
C8—C9	1.530 (2)	C23—C28	1.401 (3)
C9—C11'	1.488 (7)	C24—C25	1.379 (3)
C9—C12'	1.504 (8)	C24—H24A	0.9300
C9—C11	1.512 (6)	C25—C26	1.379 (4)
C9—C10'	1.512 (6)	C25—H25A	0.9300
C9—C12	1.531 (5)	C26—C27	1.375 (4)
C9—C10	1.542 (6)	C26—H26A	0.9300
C10—H10A	0.9600	C27—C28	1.377 (3)
C10—H10B	0.9600	C27—H27A	0.9300
C10—H10C	0.9600	C29—H29A	0.9600
C11—H11A	0.9600	C29—H29B	0.9600
C11—H11B	0.9600	C29—H29C	0.9600
C11—H11C	0.9600	C30—H30A	0.9600
C12—H12A	0.9600	C30—H30B	0.9600
C12—H12B	0.9600	C30—H30C	0.9600
C12—H12C	0.9600	C31—H31A	0.9600
C10'—H10D	0.9600	C31—H31B	0.9600
C10'—H10E	0.9600	C31—H31C	0.9600
			
O1—Al1—C31	107.63 (12)	C9—C11'—H11F	109.5
O1—Al1—C30	111.52 (11)	H11D—C11'—H11F	109.5
C31—Al1—C30	118.20 (16)	H11E—C11'—H11F	109.5
O1—Al1—N1	95.64 (6)	C9—C12'—H12D	109.5
C31—Al1—N1	112.26 (10)	C9—C12'—H12E	109.5
C30—Al1—N1	109.29 (10)	H12D—C12'—H12E	109.5
C15—N1—C16	115.41 (14)	C9—C12'—H12F	109.5
C15—N1—Al1	120.04 (11)	H12D—C12'—H12F	109.5
C16—N1—Al1	124.33 (10)	H12E—C12'—H12F	109.5
C22—N2—C21	118.35 (15)	C8—C13—C14	122.15 (15)
C1—O1—Al1	132.93 (11)	C8—C13—H13A	118.9
C28—O2—C29	118.4 (2)	C14—C13—H13A	118.9
O1—C1—C14	120.02 (15)	C13—C14—C1	120.27 (15)
O1—C1—C2	121.14 (15)	C13—C14—C15	116.88 (14)
C14—C1—C2	118.83 (15)	C1—C14—C15	122.85 (15)
C7—C2—C1	117.43 (15)	N1—C15—C14	128.32 (15)
C7—C2—C3	122.35 (15)	N1—C15—H15A	115.8
C1—C2—C3	120.22 (15)	C14—C15—H15A	115.8
C6—C3—C4	107.41 (17)	N1—C16—C17	111.59 (14)
C6—C3—C5	107.91 (17)	N1—C16—C21	109.95 (13)
C4—C3—C5	109.59 (17)	C17—C16—C21	111.44 (14)
C6—C3—C2	111.74 (15)	N1—C16—H16A	107.9
C4—C3—C2	110.11 (16)	C17—C16—H16A	107.9
C5—C3—C2	110.01 (15)	C21—C16—H16A	107.9
C3—C4—H4A	109.5	C16—C17—C18	111.44 (16)
C3—C4—H4B	109.5	C16—C17—H17A	109.3
H4A—C4—H4B	109.5	C18—C17—H17A	109.3
C3—C4—H4C	109.5	C16—C17—H17B	109.3
H4A—C4—H4C	109.5	C18—C17—H17B	109.3
H4B—C4—H4C	109.5	H17A—C17—H17B	108.0
C3—C5—H5A	109.5	C19—C18—C17	111.50 (16)
C3—C5—H5B	109.5	C19—C18—H18A	109.3
H5A—C5—H5B	109.5	C17—C18—H18A	109.3
C3—C5—H5C	109.5	C19—C18—H18B	109.3
H5A—C5—H5C	109.5	C17—C18—H18B	109.3
H5B—C5—H5C	109.5	H18A—C18—H18B	108.0
C3—C6—H6A	109.5	C20—C19—C18	111.30 (17)
C3—C6—H6B	109.5	C20—C19—H19A	109.4
H6A—C6—H6B	109.5	C18—C19—H19A	109.4
C3—C6—H6C	109.5	C20—C19—H19B	109.4
H6A—C6—H6C	109.5	C18—C19—H19B	109.4
H6B—C6—H6C	109.5	H19A—C19—H19B	108.0
C2—C7—C8	124.99 (15)	C19—C20—C21	112.01 (16)
C2—C7—H7A	117.5	C19—C20—H20A	109.2
C8—C7—H7A	117.5	C21—C20—H20A	109.2
C13—C8—C7	116.11 (15)	C19—C20—H20B	109.2
C13—C8—C9	121.34 (15)	C21—C20—H20B	109.2
C7—C8—C9	122.53 (14)	H20A—C20—H20B	107.9
C11'—C9—C12'	112.4 (6)	N2—C21—C20	110.18 (14)
C11'—C9—C11	132.2 (4)	N2—C21—C16	109.05 (14)
C11'—C9—C10'	109.4 (5)	C20—C21—C16	109.59 (14)
C12'—C9—C10'	107.4 (4)	N2—C21—H21A	109.3
C11—C9—C10'	69.8 (4)	C20—C21—H21A	109.3
C11'—C9—C12	65.0 (5)	C16—C21—H21A	109.3
C12'—C9—C12	138.2 (4)	N2—C22—C23	122.88 (18)
C11—C9—C12	110.0 (4)	N2—C22—H22A	118.6
C10'—C9—C12	46.7 (3)	C23—C22—H22A	118.6
C11'—C9—C8	111.6 (3)	C24—C23—C28	118.7 (2)
C12'—C9—C8	108.7 (3)	C24—C23—C22	121.02 (18)
C11—C9—C8	114.0 (3)	C28—C23—C22	120.3 (2)
C10'—C9—C8	107.1 (3)	C25—C24—C23	121.1 (2)
C12—C9—C8	110.5 (2)	C25—C24—H24A	119.5
C12'—C9—C10	73.2 (5)	C23—C24—H24A	119.5
C11—C9—C10	106.4 (4)	C24—C25—C26	118.9 (3)
C10'—C9—C10	141.0 (3)	C24—C25—H25A	120.5
C12—C9—C10	106.2 (4)	C26—C25—H25A	120.5
C8—C9—C10	109.4 (2)	C27—C26—C25	121.3 (2)
C9—C10—H10A	109.5	C27—C26—H26A	119.3
C9—C10—H10B	109.5	C25—C26—H26A	119.3
H10A—C10—H10B	109.5	C26—C27—C28	119.8 (3)
C9—C10—H10C	109.5	C26—C27—H27A	120.1
H10A—C10—H10C	109.5	C28—C27—H27A	120.1
H10B—C10—H10C	109.5	O2—C28—C27	124.6 (2)
C9—C11—H11A	109.5	O2—C28—C23	115.27 (19)
C9—C11—H11B	109.5	C27—C28—C23	120.2 (3)
H11A—C11—H11B	109.5	O2—C29—H29A	109.5
C9—C11—H11C	109.5	O2—C29—H29B	109.5
H11A—C11—H11C	109.5	H29A—C29—H29B	109.5
H11B—C11—H11C	109.5	O2—C29—H29C	109.5
C9—C12—H12A	109.5	H29A—C29—H29C	109.5
C9—C12—H12B	109.5	H29B—C29—H29C	109.5
H12A—C12—H12B	109.5	Al1—C30—H30A	109.5
C9—C12—H12C	109.5	Al1—C30—H30B	109.5
H12A—C12—H12C	109.5	H30A—C30—H30B	109.5
H12B—C12—H12C	109.5	Al1—C30—H30C	109.5
C9—C10'—H10D	109.5	H30A—C30—H30C	109.5
C9—C10'—H10E	109.5	H30B—C30—H30C	109.5
H10D—C10'—H10E	109.5	Al1—C31—H31A	109.5
C9—C10'—H10F	109.5	Al1—C31—H31B	109.5
H10D—C10'—H10F	109.5	H31A—C31—H31B	109.5
H10E—C10'—H10F	109.5	Al1—C31—H31C	109.5
C9—C11'—H11D	109.5	H31A—C31—H31C	109.5
C9—C11'—H11E	109.5	H31B—C31—H31C	109.5
H11D—C11'—H11E	109.5		
			
O1—Al1—N1—C15	−3.01 (14)	O1—C1—C14—C13	175.75 (15)
C31—Al1—N1—C15	−114.68 (18)	C2—C1—C14—C13	−5.0 (3)
C30—Al1—N1—C15	112.15 (17)	O1—C1—C14—C15	−5.0 (3)
O1—Al1—N1—C16	−177.28 (12)	C2—C1—C14—C15	174.21 (15)
C31—Al1—N1—C16	71.05 (17)	C16—N1—C15—C14	176.41 (15)
C30—Al1—N1—C16	−62.12 (17)	Al1—N1—C15—C14	1.7 (2)
C31—Al1—O1—C1	116.42 (19)	C13—C14—C15—N1	−178.02 (17)
C30—Al1—O1—C1	−112.4 (2)	C1—C14—C15—N1	2.8 (3)
N1—Al1—O1—C1	0.91 (18)	C15—N1—C16—C17	147.68 (15)
Al1—O1—C1—C14	2.8 (3)	Al1—N1—C16—C17	−37.81 (18)
Al1—O1—C1—C2	−176.44 (13)	C15—N1—C16—C21	−88.14 (18)
O1—C1—C2—C7	−176.44 (16)	Al1—N1—C16—C21	86.37 (15)
C14—C1—C2—C7	4.3 (2)	N1—C16—C17—C18	179.34 (15)
O1—C1—C2—C3	3.3 (3)	C21—C16—C17—C18	56.0 (2)
C14—C1—C2—C3	−175.90 (16)	C16—C17—C18—C19	−54.4 (2)
C7—C2—C3—C6	−2.0 (3)	C17—C18—C19—C20	54.0 (2)
C1—C2—C3—C6	178.25 (17)	C18—C19—C20—C21	−55.6 (2)
C7—C2—C3—C4	−121.24 (19)	C22—N2—C21—C20	116.73 (17)
C1—C2—C3—C4	59.0 (2)	C22—N2—C21—C16	−122.96 (17)
C7—C2—C3—C5	117.87 (19)	C19—C20—C21—N2	176.30 (16)
C1—C2—C3—C5	−61.9 (2)	C19—C20—C21—C16	56.3 (2)
C1—C2—C7—C8	−0.5 (3)	N1—C16—C21—N2	58.78 (17)
C3—C2—C7—C8	179.75 (16)	C17—C16—C21—N2	−176.96 (13)
C2—C7—C8—C13	−2.8 (2)	N1—C16—C21—C20	179.45 (13)
C2—C7—C8—C9	179.01 (15)	C17—C16—C21—C20	−56.28 (19)
C13—C8—C9—C11'	18.0 (8)	C21—N2—C22—C23	178.37 (15)
C7—C8—C9—C11'	−163.8 (8)	N2—C22—C23—C24	−12.8 (3)
C13—C8—C9—C12'	142.6 (6)	N2—C22—C23—C28	167.42 (17)
C7—C8—C9—C12'	−39.3 (6)	C28—C23—C24—C25	−0.6 (3)
C13—C8—C9—C11	−176.7 (6)	C22—C23—C24—C25	179.60 (19)
C7—C8—C9—C11	1.5 (6)	C23—C24—C25—C26	0.5 (3)
C13—C8—C9—C10'	−101.7 (6)	C24—C25—C26—C27	−0.3 (4)
C7—C8—C9—C10'	76.5 (6)	C25—C26—C27—C28	0.2 (4)
C13—C8—C9—C12	−52.3 (5)	C29—O2—C28—C27	0.4 (3)
C7—C8—C9—C12	125.9 (5)	C29—O2—C28—C23	−178.89 (19)
C13—C8—C9—C10	64.3 (5)	C26—C27—C28—O2	−179.6 (2)
C7—C8—C9—C10	−117.6 (5)	C26—C27—C28—C23	−0.4 (3)
C7—C8—C13—C14	2.1 (2)	C24—C23—C28—O2	179.87 (18)
C9—C8—C13—C14	−179.66 (15)	C22—C23—C28—O2	−0.3 (3)
C8—C13—C14—C1	1.7 (3)	C24—C23—C28—C27	0.5 (3)
C8—C13—C14—C15	−177.54 (15)	C22—C23—C28—C27	−179.66 (19)
